# Capacity and site readiness for hypertension control program implementation in the Federal Capital Territory of Nigeria: a cross-sectional study

**DOI:** 10.1186/s12913-021-06320-8

**Published:** 2021-04-09

**Authors:** Ikechukwu A. Orji, Abigail S. Baldridge, Kasarachi Omitiran, Mainzhao Guo, Whenayon Simeon Ajisegiri, Tunde M. Ojo, Gabriel Shedul, Namratha R. Kandula, Lisa R. Hirschhorn, Mark D. Huffman, Dike B. Ojji

**Affiliations:** 1 Cardiovascular Research Unit, University of Abuja Teaching Hospital, Gwagwalada, Abuja, Nigeria; 2 Northwestern University Feinberg School of Medicine, Chicago, IL USA; 3 The George Institute for Global Health, University of New South Wales, Sydney, Australia; 4 University of Abuja, Abuja, Nigeria

**Keywords:** Hypertension, Nigeria, Primary health care, Capacity, Readiness

## Abstract

**Background:**

Nigeria faces an increase in the burden of non-communicable diseases (NCDs), including cardiovascular diseases (CVDs), leading to an estimated 29% of all deaths in the country. Nigeria has an estimated hypertension prevalence ranging from 25 to 40% of her adult population. Despite this high burden, awareness (14–30%), treatment (< 20%), and control (9%) rates of hypertension are low in Nigeria. Against this backdrop, we sought to perform capacity and readiness assessments of public Primary Healthcare Centers (PHCs) to inform Nigeria’s system-level hypertension control program’s implementation and adaptation strategies.

**Methods:**

The study employed a multi-stage sampling to select 60 from the 243 PHCs in the Federal Capital Territory (FCT) of Nigeria. The World Health Organization (WHO) Service Availability and Readiness Assessment was adapted to focus on hypertension diagnosis and treatment and was administered to PHC staff from May 2019 – October 2019. Indicator scores for general and cardiovascular service readiness were calculated based on the proportion of sites with available amenities, equipment, diagnostic tests, and medications.

**Results:**

Median (interquartile range [IQR]) number of full-time staff was 5 (3–8), and were predominantly community health extension workers (median = 3 [IQR 2–5]). Few sites (*n* = 8; 15%) received cardiovascular disease diagnosis and management training within the previous 2 years, though most had sufficient capacity for screening (*n* = 58; 97%), diagnosis (*n* = 56; 93%), and confirmation (*n* = 50; 83%) of hypertension. Few PHCs had guidelines (*n* = 7; 13%), treatment algorithms (*n* = 3; 5%), or information materials (*n* = 1; 2%) for hypertension. Most sites (*n* = 55; 92%) had one or more functional blood pressure apparatus. All sites relied on paper records, and few had a functional computer (*n* = 10; 17%) or access to internet (*n* = 5; 8%). Despite inclusion on Nigeria’s essential medicines list, 35 (59%) PHCs had zero 30-day treatment regimens of any blood pressure-lowering medications in stock.

**Conclusions:**

This first systematic assessment of capacity and readiness for a system-level hypertension control program within the FCT of Nigeria demonstrated implementation feasibility based on the workforce, equipment, and paper-based information systems, but a critical need for essential medicine supply strengthening, health-worker training, and protocols for hypertension treatment and control in Nigeria.

## Background

Nigeria faces an increase in the burden of non-communicable diseases (NCDs), causing an estimated 29% of all deaths in Nigeria, including 22% of premature deaths [1, 2]. Moreover, more than one-third of all NCD-related deaths in Nigeria are due to cardiovascular diseases (CVDs) [1]. Meanwhile, hypertension is the leading modifiable risk factor for CVD-related morbidity and mortality in Nigeria [2]. The estimated prevalence of hypertension among adults in Nigeria, defined as blood pressure (BP) of 140/90 mmHg or higher or taking one or more BP-lowering drug(s), ranges from 25 to 40% of adults [3]. Despite this high burden, hypertension awareness (14–30%), treatment (< 20%), and control (9%) rates are low [2]. Newer definitions of hypertension based on lower blood pressure thresholds raise these hypertension prevalence estimates even higher [4].

With guidance by the WHO Tools for National Multi-sectoral Action Plan for prevention and control of Non-Communicable Diseases (NCD MAP Tool), and in line with the United Nations targets for NCDs, the Nigerian government, through the National Multi-Sectoral Action Plan for the Prevention and Control of Non-Communicable Diseases (NMAP), set national goals for NCDs. These include reducing the risk of premature (30–69 years) mortality from NCDs, including CVDs, by 25% by 2025 [5, 6].

The country will not achieve this target without substantially improving BP control by providing hypertensive care services through Nigeria’s primary health care system, which comprise about 88% of health facilities in Nigeria, where most of the populace receive their care [7]. Primary Healthcare Centers (PHCs) in low- and middle-income countries (LMICs) frequently lack system capacity for hypertension screening, diagnosis, registration of diagnosed patients, follow-up, provision of essential drugs, and treatment protocols for hypertension management [8]. In Nigeria, as of 2017, only 30% of PHCs reported availability of essential NCDs medicines, no PHCs had CVD management guidelines, and none offered CVD risk stratification services [9]. The World Health Organization (WHO) 2018 HEARTS Technical Package provides guidance for PHCs on hypertension training, diagnosis, treatment protocols, and monitoring systems to address these NCD gaps, based on the Kaiser Permanente Northern California model of hypertension care, which increased hypertension control from 44 to 90% in the United States [10, 11].

In response to these needs and context, the Hypertension Treatment in Nigeria Program (NCT04158154) [12] aims to develop implementation pathways and intervention packages for a system-level, large-scale hypertension program adapted from WHO’s HEARTS and the Kaiser Permanente Northern California model to improve hypertension diagnosis, treatment, and control rates among patients attending public PHCs in the Federal Capital Territory of Nigeria [10, 11]. During the formative phase of this Program, we performed facility-based capacity and readiness assessments among participating PHCs in the Federal Capital Territory of Nigeria to inform the implementation and adaptation of strategies for a system-level hypertension control program. These activities were implemented in collaboration with key partners, including the Federal Ministry of Health in Nigeria, Federal Capital Territory Primary Health Care Board, WHO Nigeria office, and Resolve to Save Lives.

## Methods

### Survey adaptation

This formative study used an adaptation of the Service Availability and Readiness Assessment (SARA) tool to assess 60 PHCs across the six area councils of the Federal Capital Territory in Nigeria (the adapted tool is available at doi:10.18131/g3-rknh-rr75). The SARA tool was developed by the WHO to assess health facilities’ availability and readiness to diagnose and treat common medical problems. Data collected with this tool can help in planning and managing health systems [13, 14]. It generates a set of core indicators on the health system’s critical inputs and outputs to measure health system capacity over time. There are three main domains: 1) service availability, 2) general service readiness, and 3) service-specific readiness [14].

The research team adapted the SARA tool with input from the NCD divisions of the WHO Nigeria, Nigeria Federal Ministry of Health, and other relevant stakeholders, including the Federal Capital Territory Primary Health Care Board and Federal Capital Territory Public Health Department, to focus on non-communicable diseases diagnosis, treatment, and management, specifically hypertension and diabetes mellitus. The SARA tool has 13 sections: 1) service availability, 2) patient access, 3) staffing capacity, 4) infrastructure, 5) basic client amenities, 6) infection control, 7) healthcare waste management, 8) clinical mentoring, 9) basic equipment, 10) available services for non-communicable diseases and diagnostics, 11) supply chain, 12) medicines and vaccines, and 13) commodities.

### Site selection

We identified all (*n* = 243) public PHCs within the six council areas and 62 wards in Nigeria’s Federal Capital Territory. Health facility-level characteristics, including the number of staff, the cadre of staff, the mean number of hypertension cases per month, and the number of bed spaces (if applicable), were collected from each PHC. Facilities with a ward focal person (i.e., a community health extension worker with a supervisory role over other PHCs in the ward) and facilities receiving the Primary Health Care Board’s basic healthcare provision funds from the federal government were enumerated to account for these factors in the sample selection process. These sites are the largest PHCs in terms of human resources and patient volume, and as such, were most likely to adopt the study procedures.

**Fig. 1:**
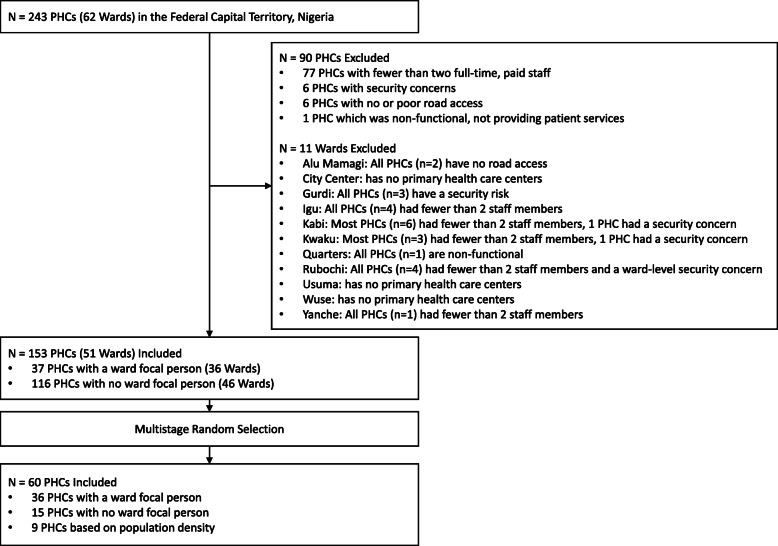
STROBE Site Flow Chart. Of 243 Primary Healthcare Centers within the Federal Capital Territory, Abuja, Nigeria, 90 were excluded based on having fewer than two full-time staff, security concerns, no or poor road access or lack of functionality by providing patient services at the time of the assessment. Of the remaining 153 Primary Healthcare Centers, multistage random selection was applied to select 60 for inclusion in the study

**Fig. 2:**
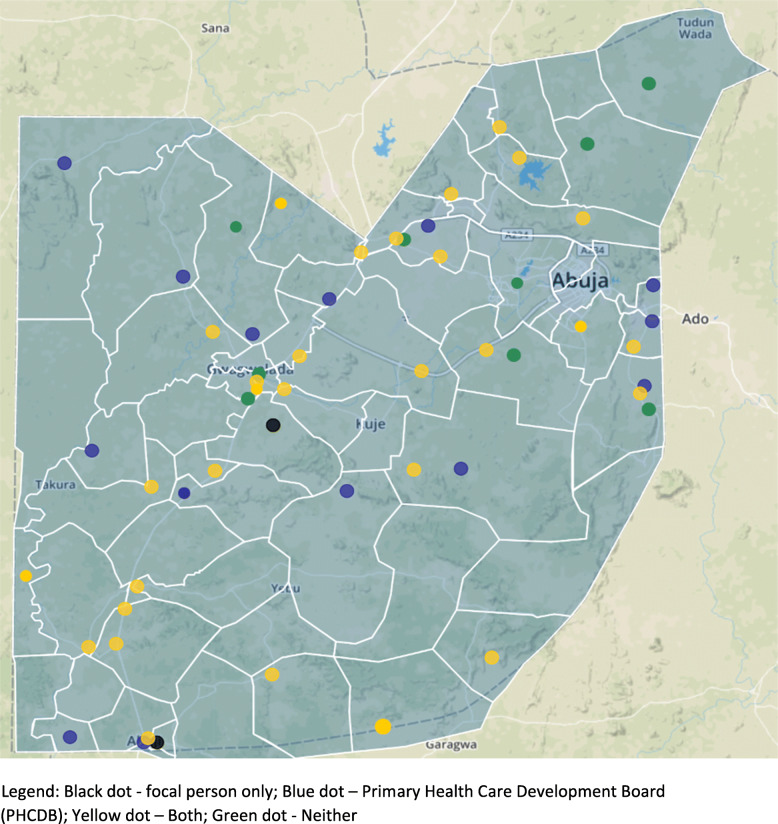
Selected Primary Healthcare Centers within the Federal Capital Territory. The 60 Primary Healthcare Centers selected for the study represent broad geographic diversity within the Federal Capital Territory, depicted here with wards represented in black lines. Some of the selected sites (black) have a ward-level focal person based within the site, and some (blue) are sites of interest for the FCT Primary Health Care Board for Basic Health Care Provision Fund (BHCPF) project. Many sites (yellow) are both

Consistent with the SARA methodology, a sub-nationally representative sample of PHCs was selected using a multi-stage sampling process (Fig. 1) [15]. Some PHCs (*n* = 90) were excluded from the sample based on the feasibility of study implementation and evaluation. These include the PHCs that had fewer than two paid full-time staff prior to study initiation based on local knowledge of PHC staffing data (*n* = 77); and the ones associated with security concerns based on local knowledge (*n* = 6); also, those with no or poor road access (*n* = 6), and the PHCs that were non-functional defined by lack of provision of patient services at the time of assessment (*n* = 1). The PHCs with less than two full time staff were excluded because such PHCs would not be likely to adopt the study procedures, in addition to their routine clinical and community work. The remaining eligible PHCs (*n* = 153) represented 51 wards within the Federal Capital Territory (Fig. 2).

The research team performed the multi-stage sampling process using SAS proc. survey select, with sampling weights for each of the 153 eligible PHCs proportional to the estimated mean number of patients with hypertension seen at each PHC per month. The sampling process included three steps: 1) identification of sites (*n* = 37 PHCs) that housed a ward focal person (i.e., a healthcare worker with a supervisory responsibility across the ward), and one PHC with a ward focal person from each ward was randomly sampled (*n* = 36 PHCs in 36 wards); 2) within the remaining 15 wards (*n* = 38 PHCs) with no ward focal person in any PHC, one site per ward was randomly sampled (*n* = 15 PHCs, in 15 wards); and 3) within the remaining PHCs (*n* = 102 PHCs), the team randomly sampled sites from the Abuja Municipal (*n* = 6 PHCs), Gwagwalada (*n* = 2 PHCs), and Kuje (*n* = 1 PHCs) area councils to approximate geographic representation by population density.

The researchers contacted selected sites to confirm their willingness to participate in the Hypertension Treatment in Nigeria Program, including a baseline SARA assessment and interview; all (100%) sites confirmed willingness to participate. They travelled to each selected PHC to invite and obtain written informed consent from the highest-level site staff or officer-in-charge and unit heads of nursing, pharmacy, and laboratory domains, to perform interviews and site assessment. The overall assessment included directly observing operations, equipment, medications, and supplies present at the site on the day of the interview. The research team completed all SARA interviews by speaking directly with the site staff to respond to survey questions.

#### Definition of key indicators

Personnel were defined as reported full-time clinicians or paramedics, nursing professionals, pharmacists, laboratory technicians, community health extension workers, and community health officers. Service availability and readiness was defined as the proportion of amenities, equipment, diagnostic tests, or medicines within a defined domain of the data collection instrument. Cardiovascular disease service availability was defined as the proportion of facilities offering cardiovascular disease diagnostic or treatment services.

### Statistical analyses

The study used graphical representations to assess the facility-based capacity and readiness for hypertension diagnosis and treatment. Continuous measures were summarized by mean and standard deviation, or median and interquartile range if non-parametrically distributed. Readiness and capacity were assessed based on domains of interest, including personnel, general service delivery, and cardiovascular service delivery in the hypertension treatment cascade, equipment and supplies, information systems, and blood pressure-lowering medications. Indicator scores for general and cardiovascular service readiness were calculated based on the proportion of sites with available amenities, equipment, diagnostic tests, or medicines within the SARA-defined domain. For statistical analysis, the study team used SAS version 9.4 (SAS, Cary, NC, USA) and R version 3.5.1 (R Foundation, Vienna, Austria).

## Results

### Participants

The team completed SARA assessments at all (*n* = 60) PHCs between May 2019 – October 2019. Among 60 participating PHCs, 36 had a ward focal person, and 34 were sites of interest to the Federal Capital Territory Primary Health Care Board as target facilities for investments to achieve universal health coverage.

### Staff and service delivery

Staffing levels, based on full or part-time status, and service delivery were tabulated based on interviews with the officer in charge. Most PHCs (*n* = 54; 90%) had sufficient human resource capacity according to the self-report of two or more full-time staff at the time of data collection (Table 1). The median (interquartile range [IQR]) number of full-time staff was 5 (3–8), and predominantly comprised community health extension workers (CHEWs; median = 3; IQR 2–5) and nurses (median = 1; IQR 0–2). Few (*n* = 8; 15%) sites received any training to diagnose and manage cardiovascular diseases within the previous 2 years. Forty-two (70%) sites had at least 1 nurse as a staff member. Nearly all PHCs (*n* = 58, 97%) had sufficient capacity for screening, and most had capacity for diagnosis (*n* = 56; 93%) and confirmation (*n* = 50; 83%) of hypertension. Over half had capacity for dispensing initial (*n* = 34; 57%) or follow-up (*n* = 34; 57%) blood pressure-lowering medications and for providing long-term continued care (*n* = 36; 60%) for patients with hypertension.

**Table 1:** Capacity and Readiness in Federal Capital Territory, Nigeria, for Implementing System-Level Hypertension Control Program within 60 Primary Healthcare Centers

Site Characteristics	No. Sites Responded	Result
Personnel and Training		
Sites with two or more full-time staff, ^a^ n (%)	54	54 (90)
Number of full-time healthcare professionals, median (IQR)	60	5 (3–8)
Full-time community health extension workers, median (IQR)	60	3 (2–5)
Full-time nurses, median (IQR)	60	1 (0–2)
Full-time doctors (generalists and specialists), median (IQR)	60	0 (0–0)
Received CVD training within the past 2 years, n (%)	55	8 (15)
Hypertension Service Delivery		
Screen for hypertension status, n (%)	60	58 (97)
Diagnose hypertension, n (%)	60	56 (93)
Confirm hypertension diagnosis, n (%)	60	50 (83)
Dispense initial treatment for hypertension, n (%)	60	34 (57)
Dispense follow-up treatment for hypertension, n (%)	60	34 (57)
Monitor patients with hypertension, n (%)	60	48 (80)
Provide long term care for patients with hypertension, n (%)	60	36 (60)
Equipment and Supplies for Hypertension		
Guidelines, n (%)	55	7 (13)
Treatment algorithms, n (%)	55	3 (5)
Information, education, and communication, n (%)	55	1 (2)
Functional blood pressure apparatus, n (%)	60	55 (92)
Information Systems		
Use of electronic patient records, n (%)	60	0 (0)
Functional landline phone, n (%)	60	13 (22)
Functional cellular phone, n (%)	60	29 (48)
Functional computer, n (%)	60	10 (17)
Access to email or internet, n (%)	60	5 (8)
Availability of Blood Pressure Lowering Medications		
Angiotensin Converting Enzyme Inhibitor, n (%)	59	10 (17)
Angiotensin Receptor Blocker, n (%)	59	3 (5)
Beta Blocker, n (%)	59	5 (8)
Calcium Channel Blocker, n (%)	59	19 (32)
Central acting agent, n (%)	59	11 (19)
Fixed Dose Combinations, n (%)	59	4 (7)
Diuretic,^b^ n (%)	59	15 (25)
Vasodilator, n (%)	59	4 (7)
Number of 30-day treatment regimens in stock, median (IQR)	59	0 (0–20)
No 30-day treatment regimens in stock, n (%)	59	35 (59)

*CVD* Cardiovascular Disease, *IQR* Inter-Quartile Range

^a^Including all reported full-time clinicians or paramedics, nursing professionals, pharmacists, laboratory technicians, community health extension workers, and community health officers

^b^Including furosemide, spironolactone, thiazide or other diuretic

**Fig. 3:**
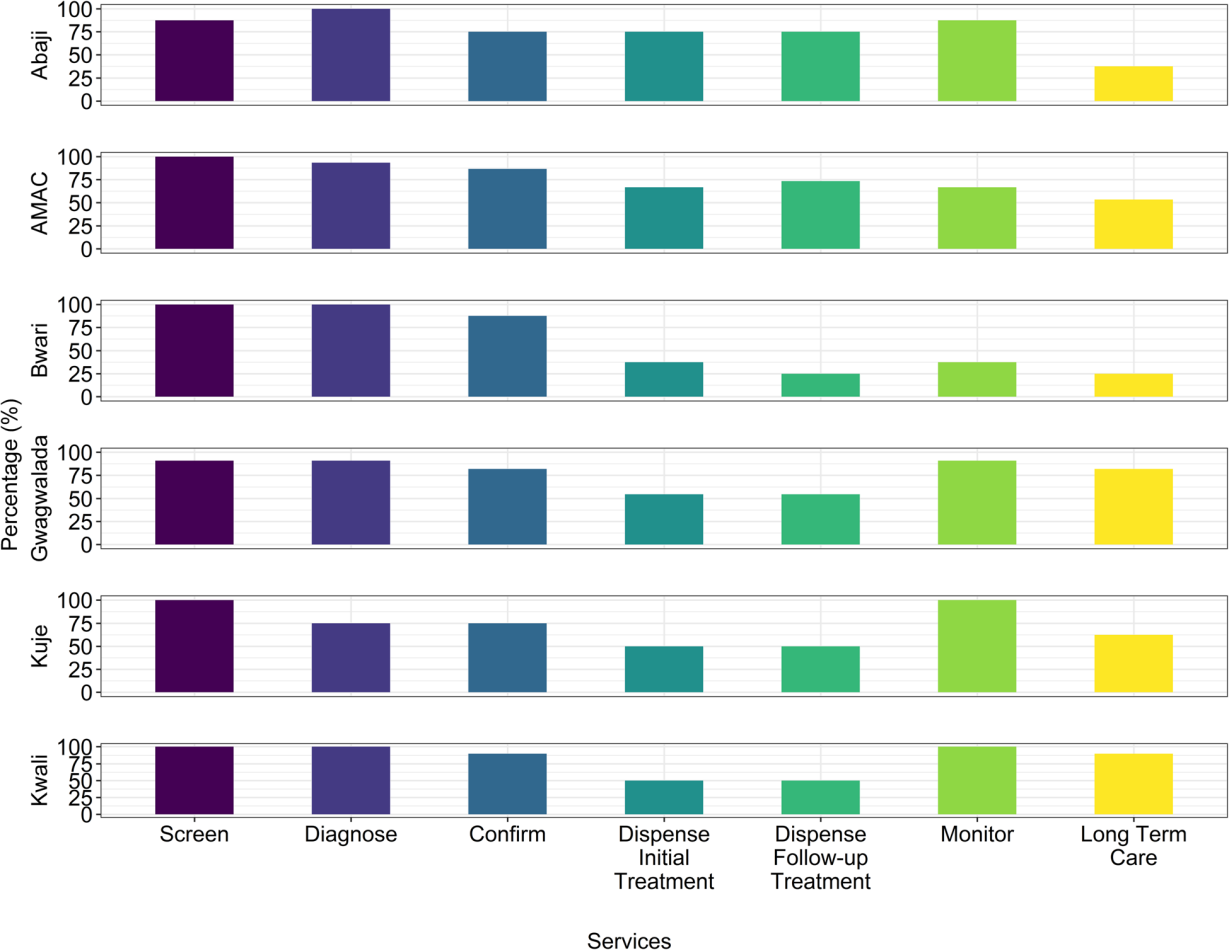
Hypertension Treatment Cascade by Council Area. Steps within the hypertension treatment cascade are shown along the x-axis, including screening, diagnosis, confirmation, treatment at initial diagnosis and at follow-up, monitoring and long-term continued care services. The proportion of primary healthcare centers within each area council who self-reported providing these services are shown by bars. Diagnosis: high blood pressure (> 140/90 mmHg) after measuring two or three times at 1–2 min intervals preceded by 3–5 min rest. Confirmation: defined as persistent high blood pressures (> 140/90 mmHg) after two or three clinic visits at 1–4 weeks intervals. Dispense initial treatment: occurs at the first visit, when a patient who has been confirmed as hypertensive is given the first 1-month course of treatment. Dispense follow-up treatment: occurs during routine monthly follow-up visit. Long term care: follow-up of patient’s treatment over several months to years

Figure 3 demonstrates the hypertension treatment cascade components across the Federal Capital Territory’s six area councils, from screening and diagnosis to monitoring and long-term care. Among the components, the highest rates were related to screening and diagnosis across all area councils and were highest in Bwari. The lowest rates were related to dispensing initial treatment regimen, follow-up treatment, and long-term care overall, with the lowest rates in Bwari.

### General and cardiovascular service readiness

Across the six area councils within the FCT, variability in general service readiness indicator scores for basic amenities, equipment, infection prevention, diagnostic capacity, and essential medicines were found (Table 2). Cardiovascular service availability was consistently high within the six area councils of the Federal Capital Territory (i.e., Abaji, Abuja Municipal Area Council [AMAC], Bwari, and Gwagwalada wards); all the PHCs reported offering cardiovascular disease diagnosis and management services. However, the study demonstrated wide variability in cardiovascular service readiness indicator scores for the presence of guidelines, equipment, and medicines. The Bwari area council consistently had the highest indicator score across general and cardiovascular domains, ranging from 29.2% for general essential medicines to 100% for equipment and cardiovascular service availability. Essential cardiovascular medicines scores, based on the availability of calcium channel blockers, aspirin, beta-blockers, ACE-I, statins, or thiazides, were low in comparison to other domains and were very low for hypertension medicines.

**Table 2:** General and Cardiovascular Disease Service Availability and Readiness Indicators for 60 Primary Healthcare Center in the Federal Capital Territory, Nigeria

Service Availability and Readiness Indicator^**a**^	Local Government Area Council					
	Abaji (***n*** = 8)	AMAC (***n*** = 15)	Bwari (***n*** = 8)	Gwagwalada (***n*** = 11)	Kuje (***n*** = 8)	Kwali (***n*** = 10)
General Service Readiness						
Basic Amenities^b^	52.1	48.9	56.3	43.9	41.7	28.3
Basic Equipment^c^	67.5	80.0	100.0	70.9	57.5	74.0
Infection Prevention^d^	93.8	56.7	81.3	68.2	75.0	90.0
Diagnostic Capacity^e^	59.4	83.3	90.6	61.4	62.5	62.5
Essential Medicines^f^	6.3	24.4	29.2	13.6	6.3	5.0
Cardiovascular Disease Service Availability						
Availability^g^	100.0	100.0	100.0	100.0	87.5	90.0
Cardiovascular Disease Readiness Indicators						
Guidelines^h^	12.5	6.7	0.0	0.0	0.0	20.0
Equipment^i^	70.9	84.4	100	69.7	66.7	73.4
Medicines^j^	7.5	28.0	30.0	16.4	7.5	6.0

^a^Each indicator is calculated as the proportion of amenities, equipment, diagnostic tests, or medicines within a defined SARA domain

^b^The item “Room with auditory and visual privacy for patient consultations” was not included

^c^The item “Child scale” was not included

^d^The items “Safe final disposal of infectious wastes”, “Appropriate storage of sharps waste”, “Appropriate storage of infectious waste”, “Disinfectant”, “Single use —standard disposable or auto-disable syringes”, “Soap and running water or alcohol-based hand rub” and “Latex gloves” were not included

^e^The items “Malaria diagnostic capacity”, “HIV diagnostic capacity”, “Syphilis rapid test” and “Urine test for pregnancy” were not included

^f^Items “CCB”, “Aspirin”, “Beta Blockers”, “ACE”, “Statin” and “Thiazide” were included

^g^Calculated as the proportion of facilities offering cardiovascular disease diagnosis and/or management

^h^The item “guidelines for diagnosis and treatment of chronic cardiovascular conditions” was included

^i^The items “Stethoscope”, “Blood pressure apparatus” and “Adult scale” were included

^j^The items “CCB”, “Aspirin”, “Beta Blockers”, “ACE” and “Thiazide” were included

### Equipment, supplies, & information systems

Equipment, supplies, and information systems were assessed in consultation with the officer-in-charge and staff in charge of medical records and data and based on direct observation to determine the availability and function of equipment on the day of assessment. Few PHCs had guidelines (*n* = 7; 13%), treatment algorithms (*n* = 3; 5%), or information materials (*n* = 1; 2%) for hypertension diagnosis or management within the clinic on the day of assessment. Most sites (*n* = 55; 92%) had at least one functional BP apparatus present. All sites relied on paper-based longitudinal records, and relatively few had a functional computer (*n* = 10; 17%) or access to the internet or email (*n* = 5; 8%).

### Medications

**Fig. 4:**
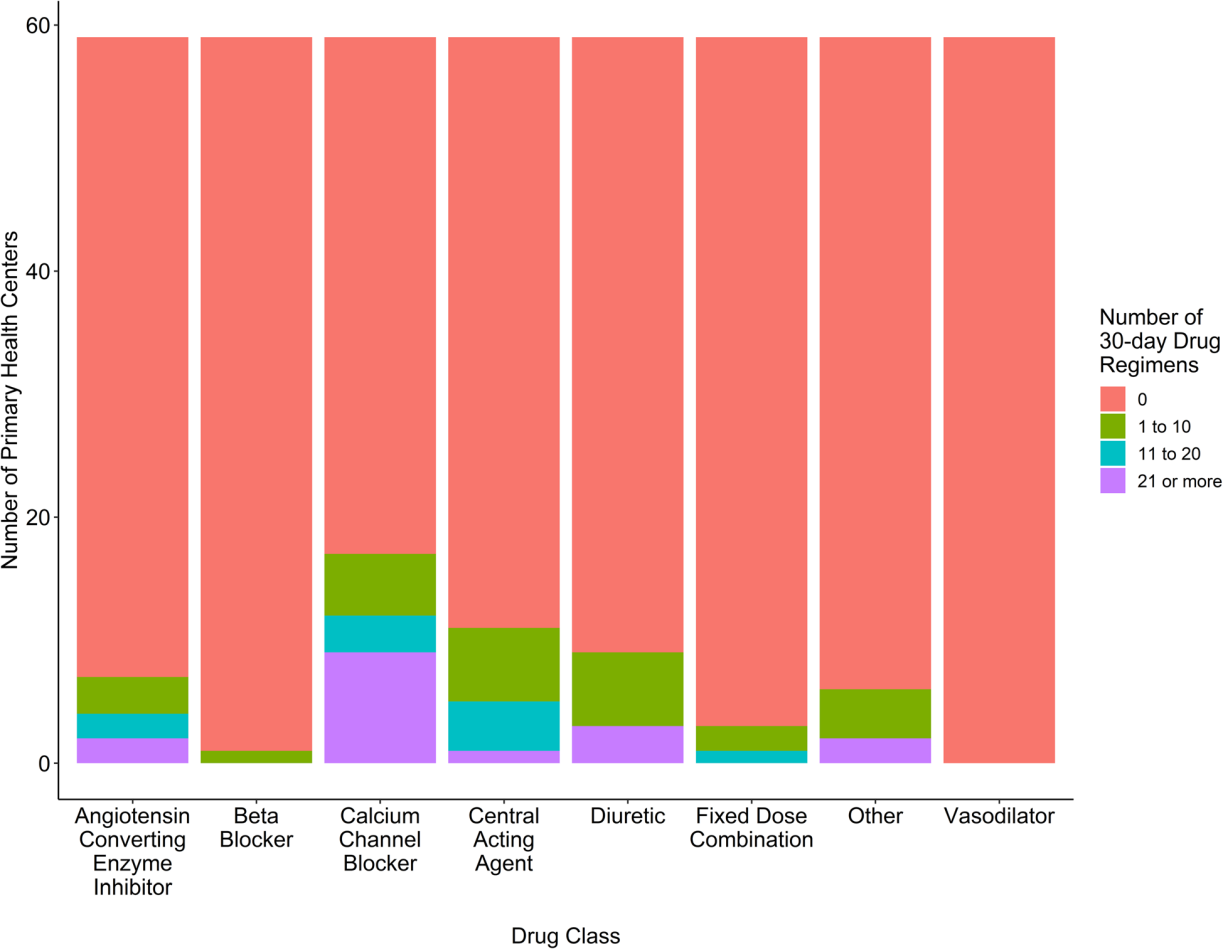
Drugs Available for 30-Day Regimens by Drug Class among Selected PHCs (*n* = 59). The number of 30-day treatment regimens in stock of the day of assessment were tabulated by drug class. Most sites had no 30-day treatment regimens in stock. Of the drugs that were stocked, calcium channel blockers, central acting agents, diuretics and angiotensin converting enzymes inhibitors (ACE-I) were most common

Hypertension medications were tabulated based on direct observation of the pharmaceutical inventory against record logs, where available. The adapted SARA tool classified medications into broad classes. Figure 4 reports the number of 30-day treatment regimens available on the day of assessment by class. At one site, the pharmaceutical inventory was not accessible on the day of the visit. The most commonly stocked medications were calcium channel blockers (*n* = 19; 32%), followed by diuretics (*n* = 15; 25%), central acting agents (*n* = 11; 19%), and angiotensin-converting enzyme inhibitors (ACE-I; *n* = 10; 17%). Despite their inclusion on the WHO and Nigeria essential medicines lists, few PHCs (*n* = 4; 7%) had fixed-dose combinations for hypertension treatment. The median (IQR) number of 30-day treatment regimens of all blood pressure-lowering medications in stock on the day of assessment was 0 (0–20) regimens, and 35 (59%) PHCs had zero 30-day treatment regimens of BP-lowering medication in stock.

## Discussion

While Nigeria has set a national goal of reducing the risk of premature (30–69 years) mortality from NCDs, including CVDs, by 25% by 2025 [ 5, 6], the country has not fully translated into action within the PHCs the necessary components to fully implement system-level hypertension services. Although cardiovascular service availability is high in the Federal Capital Territory, there is wide variability in readiness to provide these services identified, including the presence of guidelines, equipment, and medicines. We demonstrated the feasibility of implementing the Hypertension Treatment in Nigeria Program based on the workforce, equipment, and paper-based information systems, but identified critical needs for health worker training, protocol implementation, and essential medicine supply strengthening for hypertension treatment and control.

### Staff and service delivery

Our study shows that full-time staff members were primarily CHEWs and nurses, who comprise the cadre of non-physician health workers needed to provide service delivery in community-based care. PHCs need qualified, trained staff to implement an effective task-shifting strategy for improved NCDs management, including hypertension [16]. Our benchmark of including PHCs with at least two full-time staff as sufficient human resource capacity for implementing the Hypertension Treatment in Nigeria Program aligns closely with the minimum number of CHEWs required to operate a PHC [17]. The relatively large number of non-physician health workers in PHCs in this study, including CHEWs, may be partially attributed to the cumulative density of schools of health technology, which are training institutions for CHEWs in Nasarawa, Kogi, Kaduna, and Niger states, that share boundary with the Federal Capital Territory [18]. This contrasts with the 2018 report from Garcia de Quevedo et al., who identified limited NCDs workforce as a challenge in Mozambique, Colombia, and the Dominican Republic based on the high rate of temporary contract workers for NCD-related care [19]. Moucheraud reported low staff and training readiness scores for NCD diagnosis and management using Service Provision Assessment surveys across PHCs in Bangladesh (24%), Haiti (29%), Malawi (18%), Nepal (4%), and Tanzania (12%) [20], signifying that low staff and service delivery readiness is common throughout LMICs. These differences between the current study and data reported by Garcia de Quevedo et al. and Moucheraud are due, at least in part, to the current study’s inclusion criterion of PHCs with at least two full-time health workers for Hypertension Treatment in Nigeria Program implementation. While staff in the current study were integrated into the broader PHC delivery system in Nigeria, they also had responsibilities beyond hypertension and NCDs.

We identified substantial gaps in training for diagnosing and managing CVDs, including gaps in standing orders for CHEW-based CVD- and hypertension-related care, unlike other chronic conditions such as human immunodeficiency virus (HIV). Lack of adequate staff training is a recurring challenge in health program implementation in LMICs [21–23]. These results inform initial training and longitudinal re-training activities before and during the Hypertension Treatment in Nigeria Program, given the fundamental need for training and supportive supervision. Further investments in staff and staff training and development and implementation of standing orders for managing CVD and hypertension may be needed to keep pace with these conditions’ growing burden.

### Service readiness

In terms of general service readiness, the domains of basic equipment, infection prevention, and diagnostic capacity reported across all area councils revealed that more than half of facilities met general readiness criteria. Meeting these criteria may have been influenced by federal and local government support and commitments for other disease control programs in these PHCs, including maternal and child health. In terms of basic amenities (e.g., portable water, toilet facility, power supply), only two out of six area councils had more than half of their PHCs meeting the criteria. Further, essential medicine provision indices were low across all the area councils, demonstrating the heterogeneous investments in capital-intensive infrastructure. According to Oyekale et al. [22], the effective delivery of healthcare services at the PHC system requires adequate infrastructure, diagnostic medical equipment, essential medicines, and well-trained health workers. Therefore, there is a need to strengthen service readiness for effective Hypertension Treatment in Nigeria Program implementation and service delivery.

### Equipment, supplies, & information systems

We found low availability of guidelines, treatment algorithms, and information materials for hypertension, similar to previous reports in other sub-Saharan African countries, including Tanzania [21, 23]. We found that while most sites had at least one functional BP apparatus, only a few PHCs had hypertension guidelines, treatment algorithms, information, education, and communication materials. These results also underscore the importance of Nigeria’s Federal Ministry of Health’s recent steps to integrate the WHO-recommended Package of Essential NCD interventions (PEN) into PHCs in collaboration with key stakeholders to address this gap [24]. Although few PHCs had computers or internet access available onsite with no sites reporting electronic patient records, Nigeria’s PHCs generally have a well-developed paper-based information management system for programs like HIV care, antenatal care, and family planning, which can be leveraged for the treatment of hypertension and other NCDs. Substantial investments in infrastructure, hardware, software, secure internet access, and training will be needed to upgrade to an electronic data management system to achieve the WHO’s four functions of health information systems: 1) data generation, 2) compilation, 3) analysis and synthesis, and 4) communication and use within care teams and patients [14]. Our results are similar to reports of lower availability of CVDs guidelines and higher availability of equipment for CVDs among health facilities in LMICs [20], but differ from other findings which describe lack of essential equipment as a critical barrier to providing quality NCDs services at the primary care level [21].

### Medications

We found that while most PHCs had sufficient capacity for screening, diagnosis, and confirmation of hypertension, more than half of them did not have any 30-day treatment regimens of blood pressure-lowering medicines with significant variability across area councils. This finding is similar to that of Moucheraud et al. [20], who reported that most facilities in LMICs studied had limited essential cardiovascular medicines. Low availability of cardiovascular medicines at PHCs has also been reported in other LMICs such as Tanzania (25 to 40%), Bangladesh (30%), Nepal (28%), and higher rates have been reported in Malawi (67%) and Haiti (93%) [20, 25]. Provision of essential medicines for hypertension is generally a challenge within PHC systems in sub-Saharan Africa. The scarcity of medications may be explained by a lack of government-supported programs for NCDs within PHC systems. There are opportunities for support, mainly through strengthening drug revolving funds, which rely upon initial investments and recurring, reduced out-of-pocket costs to sustain the funds and subsequent medicine supplies. Drug revolving funds may improve the availability of BP-lowering drugs in the PHCs until community insurance programs are fully funded and established [26].

### Strengths and limitations

Key strengths of the current study include 1) sampling frame to maximize generalizability in the Federal Capital Territory, 2) adaptation of the SARA instrument to hypertensive services in the local context, and 3) engagement with PHCs and other stakeholders, including the Federal Ministry of Health. This study also has significant limitations. First, the study excluded facilities with fewer than two full-time staff and is therefore not representative of all PHCs in the Federal Capital Territory or all of Nigeria, which limits generalizability. While we did not collect data from sites with less than two full-time staff, it is likely that service availability and readiness would be lower at such sites. Nevertheless, the Hypertension Treatment in Nigeria Program aims to improve hypertension treatment and control rates, and the presence of two full-time staff is necessary for sites to feasibly adopt the study procedures. Further, the SARA instrument collects cross-sectional data, and therefore temporal trends in service availability and readiness among the included PHCs were not captured. However, the study team will perform repeated cross-sectional assessments using the adapted SARA instrument throughout the Hypertension Treatment in Nigeria Program to address this limitation. The SARA instrument is also susceptible to recall and reporting biases by deriving responses from the highest-level site staff or officer-in-charge. The research team mitigated this risk by direct observation of supplies and equipment where possible. However, the BP-lowering drug availability estimates do not include the availability of essential medicines through private pharmacies, where out-of-pocket costs are often higher than public pharmacies [27], but this was beyond the direct scope of the data collected herein. Finally, measures of service availability and readiness may not capture the quality and experience of service provision, which have generally become a larger contributor to health loss in LMICs compared with service availability alone [28].

## Conclusions

This study is the first systematic assessment of capacity and readiness for a system-level hypertension control program within the Federal Capital Territory of Nigeria. We demonstrated the feasibility of implementing hypertension diagnostic and treatment services based on the workforce, equipment, and existing information systems. Nevertheless, we identified critical needs for capital intensive infrastructure investments, health worker training, hypertension treatment protocol implementation, and essential medicine supply strengthening in Nigeria. These data highlight the need for upfront and longitudinal investments across health system building blocks to deliver high-quality hypertension care at PHCs in Nigeria. These investments will be critical for Nigeria’s health system to not only respond to the current and projected burden of hypertension-related diseases but also to improve its resilience in the face of other emerging health threats.

## Data Availability

The datasets used and/or analysed during the current study available from the corresponding author on reasonable request.

## Abbreviations

AMAC: Abuja Municipal Area Council
ACE-I: Angiotensin Converting Enzyme Inhibitors
CVDs: Cardiovascular Diseases
CHEW: Community Health Extension Worker
FCT: Federal Capital Territory
HEARTS: Healthy-lifestyle counselling; Evidence-based treatment protocols; Access to essential medicines and technology; Risk-based CVD management; Team-based care; Systems for monitoring
HIV: Human Immunodeficiency Virus
IQR: Inter-Quartile Range
LMICs: Low-and Middle-Income Countries
NCDs: Non-Communicable Diseases
PEN: Package of Essential NCD interventions
PHC: Primary Healthcare Center
SARA: Service Availability and Readiness Assessment
WHO: World Health Organization

## References

[1] Reliefweb. Nigeria Fulfils Commitment, launches Plan for the Prevention and Control of Non-Communicable Diseases: United Nations Office for the Coordination of Humanitarian Affairs (OCHA) services; 2019. https://reliefweb.int/report/nigeria/nigeria-fulfils-commitment-launches-plan-prevention-and-control-non-communicable. Accessed 16 Apr 2020

[2] Ogah OS, Okpechi I, Chukwuonye II, Akinyemi JO, Onwubere BJ, Falase AO, Stewart S, Sliwa K (2012). Blood pressure, prevalence of hypertension and hypertension related complications in Nigerian Africans: a review. World J Cardiol.

[3] Adeloye D, Basquill C, Aderemi AV, Thompson JY, Obi FA (2015). An estimate of the prevalence of hypertension in Nigeria: a systematic review and meta-analysis. J Hypertens.

[4] Whelton PK, Carey RM, Aronow WS, Casey DE, Collins KJ, Dennison Himmelfarb C, DePalma SM, Gidding S, Jamerson KA, Jones DW, MacLaughlin EJ, Muntner P, Ovbiagele B, Smith SC, Spencer CC, Stafford RS, Taler SJ, Thomas RJ, Williams KA, Williamson JD, Wright JT (2018). 2017 ACC/AHA/AAPA/ABC/ACPM/AGS/APhA/ASH/ASPC/NMA/PCNA guideline for the prevention, detection, evaluation, and management of high blood pressure in adults: executive summary: a report of the American College of Cardiology/American Heart Association task force on clinical practice guidelines. J Am Coll Cardiol.

[5] Federal Ministry of Health (FMoH) Nigeria (2019). National Multi-Sectoral Action Plan for the Prevention and Control of Non-Communicable Diseases (2019–2025).

[6] Sacco RL, Roth GA, Reddy KS, Arnett DK, Bonita R, Gaziano TA, Heidenreich PA, Huffman MD, Mayosi BM, Mendis S, Murray CJL, Perel P, Piñeiro DJ, Smith SC, Taubert KA, Wood DA, Zhao D, Zoghbi WA (2016). The heart of 25 by 25: achieving the goal of reducing global and regional premature deaths from cardiovascular diseases and stroke: a modeling study from the American Heart Association and world heart federation. Circulation..

[7] Primary health care systems (PRIMASYS): case study from Nigeria. Geneva: World Health Organization; 2017. Licence: CC BY-NC-SA 3.0 IGO. Available at https://www.who.int/alliance-hpsr/projects/alliancehpsr_nigeriaprimasys.pdf?ua=1. Accessed 22 Feb 2021.

[8] World Health Organization (2020). High Blood Pressure and the Role of Primary Health Care.

[9] World Health Organization (2018). Noncommunicable Diseases (NCD) Country Profiles, Nigeria.

[10] Jaffe MG, Lee GA, Young JD, Sidney S, Go AS (2013). Improved blood pressure control associated with a large-scale hypertension program. JAMA..

[11] Jaffe MG, Young JD (2016). The Kaiser Permanente northern California story: improving hypertension control from 44 to 90% in 13 years (2000 to 2013). J Clin Hypertens (Greenwich).

[12] Transforming Hypertension Treatment in Nigeria using a Type II Hybrid, Interrupted Time Series Design. Available at https://clinicaltrials.gov/ct2/results?term=hypertension+treatment+in+Nigeria&Search=Search. Accessed 25 Feb 2021.

[13] World Health Organization (2020). Health statistics and information systems. Service availability and readiness assessment (SARA).

[14] World Health Organization. Service Availability and Readiness Assessment (SARA): an annual monitoring system for service delivery. Geneva; 2013. https://www.who.int/healthinfo/systems/SARA_Reference_Manual_Full.pdf. Accessed 16 Apr 2020

[15] Sheffel A, Karp C, Creanga AA (2018). Use of service provision assessments and service availability and readiness assessments for monitoring quality of maternal and newborn health services in low-income and middle-income countries. BMJ Glob Health.

[16] Joshi R, Alim M, Kengne AP, Jan S, Maulik PK, Peiris D, Patel AA (2014). Task shifting for non-communicable disease management in low and middle income countries--a systematic review. PLoS One.

[17] National Primary Health Care Development agency Publications: PHC Guideline (2020). Operational manual and guideline for development of primary health.

[18] Yahaya A. Full list of schools of health technology in Nigeria 2020: Nigerian infopedia; 2019. https://nigerianinfopedia.com.ng/full-list-of-schools-of-health-technology-in-nigeria. Accessed 16 Apr 2020

[19] Garcia de Quevedo I, Lobelo F, Cadena L, Soares M, Pratt M (2018). A comprehensive capacity assessment tool for non-communicable diseases in low- to middle-income countries: development and results of pilot testing. Glob Health Promot.

[20] Moucheraud C (2018). Service readiness for noncommunicable diseases was low in five countries in 2013-15. Health Aff (Millwood).

[21] Bintabara D, Mpondo BCT (2018). Preparedness of lower-level health facilities and the associated factors for the outpatient primary care of hypertension: evidence from Tanzanian national survey. PLoS One.

[22] Oyekale AS (2017). Assessment of primary health care facilities' service readiness in Nigeria. BMC Health Serv Res.

[23] Leung C, Aris E, Mhalu A, Siril H, Christian B, Koda H, Samatta T, Maghimbi MT, Hirschhorn LR, Chalamilla G, Hawkins C (2016). Preparedness of HIV care and treatment clinics for the management of concomitant non-communicable diseases: a cross-sectional survey. BMC Public Health.

[24] World Health Organization (2019). WHO and Nigerian government move to curb cardiovascular diseases.

[25] Peck R, Mghamba J, Vanobberghen F, Kavishe B, Rugarabamu V, Smeeth L, Hayes R, Grosskurth H, Kapiga S (2014). Preparedness of Tanzanian health facilities for outpatient primary care of hypertension and diabetes: a cross-sectional survey. Lancet Glob Health.

[26] Uzochukwu BS, Onwujekwe OE, Akpala CO (2002). Effect of the Bamako-initiative drug revolving fund 23 on availability and rational use of essential drugs in primary health care facilities in south-East Nigeria. Health Policy Plan.

[27] Wirtz VJ, Hogerzeil HV, Gray AL, Bigdeli M, de Joncheere CP, Ewen MA, Gyansa-Lutterodt M, Jing S, Luiza VL, Mbindyo RM, Möller H, Moucheraud C, Pécoul B, Rägo L, Rashidian A, Ross-Degnan D, Stephens PN, Teerawattananon Y, 't Hoen EFM, Wagner AK, Yadav P, Reich MR (2017). Essential medicines for universal health coverage. Lancet..

[28] Kruk ME, Gage AD, Arsenault C, Jordan K, Leslie HH, Roder-DeWan S, Adeyi O, Barker P, Daelmans B, Doubova SV, English M, Elorrio EG, Guanais F, Gureje O, Hirschhorn LR, Jiang L, Kelley E, Lemango ET, Liljestrand J, Malata A, Marchant T, Matsoso MP, Meara JG, Mohanan M, Ndiaye Y, Norheim OF, Reddy KS, Rowe AK, Salomon JA, Thapa G, Twum-Danso NAY, Pate M (2018). High- quality health systems in sustainable development goals era: time for a revolution. Lancet Glob Health.

